# Editorial for “Post COVID-19 Syndrome in Patients with Asymptomatic/Mild Form”

**DOI:** 10.3390/pathogens12020167

**Published:** 2023-01-20

**Authors:** Arad Dotan, Yehuda Shoenfeld

**Affiliations:** 1 Zabludowicz Center for Autoimmune Diseases, Sheba Medical Center, Tel-Hashomer, Ramat Gan 5265601, Israel; 2 Sackler Faculty of Medicine, Tel Aviv University, Tel Aviv 6997801, Israel

The novel severe acute respiratory syndrome coronavirus 2 (SARS-CoV-2), first reported in December 2019, has infected numerous subjects worldwide. SARS-CoV-2 is the virus that led to coronavirus disease 2019 (COVID-19), which caused one of the most widespread pandemics worldwide. Most individuals infected with SARS-CoV-2 experience a mild respiratory illness and recover without requiring treatment. Nevertheless, some became severely ill and require medical attention.

Significant time has passed since the COVID-19 pandemic outbreak began, and worldwide infection and death rates have declined significantly due to the mass vaccination programs and natural post-infection immunity. Thus, fear of the acute implications of COVID-19 have lightened. However, new long-term manifestations of SARS-CoV-2 infection are rapidly increasing; recent studies analyzing recovered COVID-19 patients demonstrated a broad spectrum of persistent symptoms. These residual symptoms that persist after recovery from acute SARS-CoV-2 infection were introduced under the novel terms: “post-COVID syndrome”, “long COVID”, and “chronic COVID-19” [1,2]. In addition, this new disorder has led to the understanding that the absence of SARS-CoV-2 following COVID-19 does not necessarily mean a full recovery [1,2,3].

The mechanism of these post-COVID syndrome (PCS) manifestations is still unclear, and there have been multiple approaches to its definition and classification. Some believe symptoms persist from the acute phase and are the consequences of COVID-19 treatments or prolonged hospitalization [4]. At the same time, some researchers advocate that PCS arises after a mild or asymptomatic infection, and not merely after COVID-19 patients are severely ill [5]. Finally, some argue that PCS is a previous pathology or disability of the individual being impacted by the acute phase of COVID-19 [6]. 

Due to the unclear criteria for PCS prevention, diagnosis, management tactics, and contributing factors, Anna Malkova et al. conducted a review analysis of publications about COVID-19 in online databases from December 2019 to September 2021 [7]. This analysis looked at research on post-COVID-19 syndrome in patients with asymptomatic and mild SARS-CoV-2 infections. The inclusion criteria were asymptomatic or mild forms of COVID-19 diagnosed with a PCR test, the development of PCS symptoms a minimum of 1 month after, and the description of the developed PCS symptoms. Unfortunately, only a small number of published papers had described the type of PCS manifestations; thus, this study could not follow the PRISMA guidelines. Nevertheless, 11 studies were reviewed, which included the sum of 13,637 SARS-CoV-2-infected patients. PCS developed among approximately 30–60% of patients with asymptomatic or mild forms of COVID-19, and the most common symptoms were fatigue, shortness of breath, cough, anosmia, and ageusia. In addition, other signs of central nervous system damage were reported, including headaches and brain fog. Furthermore, a meta-analysis that defined PCS as ranging from 14 to 110 days after the viral infection estimated that 80% of the infected patients with SARS-CoV-2 developed one or more long-term symptoms [8].

Notably, females had a higher risk of developing PCS in comparison to males, with an approximately 1.25 ratio (on average, 60% of PCS patients were females). These results highlight the immense importance of further research in sufficient therapeutic agents for PCS manifestations. In addition, current evidence suggests that the severity and mortality of COVID-19 are higher in males than in females, whereas females are at an increased risk of COVID-19 reinfection and the development of PCS [9]. These conflicting risks between the severity and mortality of COVID-19 compared to the development of PCS could be explained by the generally more aggressive immune system of females [10,11]. 

Differences between the sexes are well studied in numerous infectious diseases, as well as in response to vaccinations [9,12]. Sex-specific expression patterns of proteins that mediate virus binding and entry and divergent reactions of the immune and endocrine systems, particularly the hypothalamic–pituitary–adrenal axis, in response to acute stress might explain the higher severity of COVID-19 in men [9]. Additionally, as enhanced immunoreactivity in females provides better protection against infections, it contributes to enhanced autoreactivity, thereby contributing to the induction of more autoimmune manifestations and diseases than in males, such as SLE, multiple sclerosis, rheumatoid arthritis, and numerous others [11,13,14]. Furthermore, COVID-19 has been shown to be associated with many autoantibodies, as well as autoimmune manifestations [10,15,16,17,18]. Thus, the PCS course of the disease may have at least a partial autoimmune factor; otherwise, it would not be logical that females would be at an elevated risk. Importantly, as the severity of COVID-19 is higher in males than in females, it could be assumed that the difference in the risk of PCS is not primarily due to the severity of the COVID-19 infection.

The possible association between the described PCS symptoms and brain damage that occurs during an SARS-CoV-2 infection suggests that PCS patients commonly have a clinical presentation similar to that of encephalomyelitis/chronic fatigue syndrome (ME/CFS): severe fatigue, sleep disorders, cognitive impairments, and different manifestations of autonomic dysfunction exacerbated in physical exercise [8,10]. Furthermore, close to 20 distinct autoantibodies which target the GPCR of the nervous system and renin-angiotensin system-related molecules were found to be significantly associated with the clinical severity of COVID-19 [10]. Additionally, these autoantibodies were elevated in a subset of ME/CFS patients, specifically, the β2 adrenergic and M3 and M4 cholinergic receptor antibodies, as well as in other similar disorders [19,20,21]. Thus, autoimmune-mediated autonomic nervous system dysfunction, referred to as dysautonomia, may contribute to the disease course of PCS, which is similar to ME/CFS [10,21,22,23]. Notably, supervised and moderate physical exercise was shown to stabilize the autonomic nervous system. Thus, it may also be beneficial for PCS patients. Additionally, therapeutic options such as immunomodulatory and immunosuppressive therapy could benefit some PCS patients, whereas plasmapheresis and IVIG could be considered in severe cases [1,10,22,23]. 
